# The relationship between protein modified folding molecular network and Alzheimer’s disease pathogenesis based on BAG2-HSC70-STUB1-MAPT expression patterns analysis

**DOI:** 10.3389/fnagi.2023.1090400

**Published:** 2023-05-12

**Authors:** Xiaolong Yang, Wenbo Guo, Lin Yang, Xuehui Li, Zhengkun Zhang, Xinping Pang, Ji Liu, Chaoyang Pang

**Affiliations:** ^1^Department of Biochemistry and Molecular Biology, West China School of Basic Medical Sciences and Forensic Medicine, Sichuan University, Chengdu, China; ^2^College of Computer Science, Sichuan Normal University, Chengdu, China; ^3^West China School of Basic Medical Sciences and Forensic Medicine, Sichuan University, Chengdu, China

**Keywords:** Alzheimer disease, gene expression level, tau, BAG2, HSC70, STUB1, MAPT

## Abstract

**Background:**

Alzheimer’s disease (AD) is the most common cause of dementia and cognitive decline, while its pathological mechanism remains unclear. Tauopathies is one of the most widely accepted hypotheses. In this study, the molecular network was established and the expression pattern of the core gene was analyzed, confirming that the dysfunction of protein folding and degradation is one of the critical factors for AD.

**Methods:**

This study analyzed 9 normal people and 22 AD patients’ microarray data obtained from GSE1297 in Gene Expression Omnibus (GEO) database. The matrix decomposition analysis was used to identify the correlation between the molecular network and AD. The mathematics of the relationship between the Mini-Mental State Examination (MMSE) and the expression level of the genes involved in the molecular network was found by Neural Network (NN). Furthermore, the Support Vector Machine (SVM) model was for classification according to the expression value of genes.

**Results:**

The difference of eigenvalues is small in first three stages and increases dramatically in the severe stage. For example, the maximum eigenvalue changed to 0.79 in the severe group from 0.56 in the normal group. The sign of the elements in the eigenvectors of biggest eigenvalue reversed. The linear function of the relationship between clinical MMSE and gene expression values was observed. Then, the model of Neural Network (NN) is designed to predict the value of MMSE based on the linear function, and the predicted accuracy is up to 0.93. For the SVM classification, the accuracy of the model is 0.72.

**Conclusion:**

This study shows that the molecular network of protein folding and degradation represented by “BAG2-HSC70-STUB1-MAPT” has a strong relationship with the occurrence and progression of AD, and this degree of correlation of the four genes gradually weakens with the progression of AD. The mathematical mapping of the relationship between gene expression and clinical MMSE was found, and it can be used in predicting MMSE or classification with high accuracy. These genes are expected to be potential biomarkers for early diagnosis and treatment of AD.

## 1. Introduction

Alzheimer’s Disease (AD), the most common cause of senile dementia, is a progressive neurodegenerative disorder that its prevalence is increasing substantially worldwide (Tanzi and Bertram, 2005; Singh et al., 2022). With around 50 million people suffering from this disease, no cure or preventative therapy is available (Pang et al., 2019; Silvestro et al., 2022). According to the statistics, the number of affected people will increase to 65.7 million in 2030 and 115.4 million in 2050 (Prince et al., 2013).

Tau is a member of microtubule associated protein (MAP) family, mainly located in brain axon neuron cells. Its function is to stabilize axon microtubules, and phosphorylation can reduce its ability to bind microtubules (Laurent and Blum, 2018). Native Tau is disordered and needs to be folded into a suitable motif to perform its function (Cehlar et al., 2021). Protein folding is a complicated process that involves correctly folding and stabilizing amino acid chains into functional proteins (Heneka et al., 2015). Protein misfolding can result from genetic mutations, aging, or environmental stressors, and these misfolded proteins can then aggregate into oligomers and fibrils (Zhang et al., 2021). This aggregation can lead to the formation of amyloid plaques and other protein aggregates (Kadavath et al., 2015; Pinheiro and Faustino, 2019). The abnormal folding and degradation of Tau protein have been linked to several neurodegenerative diseases, and chaperone proteins play a crucial role in the proper folding, trafficking, and intermediate stabilization of Tau (Wolfe, 2012; Pîrşcoveanu et al., 2017). In Alzheimer’s disease, misfolded beta-amyloid and Tau proteins form oligomers and fibrils that disrupt normal cellular function and activate inflammatory responses, ultimately resulting in neuronal death and brain damage (Ashrafian et al., 2021; Crestini et al., 2022).

The histopathological features of AD are neurofibrillary tangle (NFT), loss of neurons and cognitive function (Grøntvedt et al., 2018; Laurent and Blum, 2018; Lynch, 2020). Tau hyperphosphorylation leading to NFT was found in Alzheimer’s disease (Dregni et al., 2022). The formation of NFT is thought to be driven by protein misfolding and aggregation, which can disrupt normal cellular processes and lead to neuronal dysfunction and death. Specific branch research directions include hyperphosphorylation Tau (Silvestro et al., 2022), phosphoryd plasma Tau (Pilotto et al., 2022), abnormal aggregation of Tau (Gao et al., 2018), regulation of iron accumulation Tau (Rao and Adlard, 2018) and truncation Tau (Quintanilla et al., 2020). The exact mechanism of Tau leading to NFT is still controversial, but researchers cannot deny that the degradation and folding of Tau is the key point of this hypothesis (Laurent and Blum, 2018; Cehlar et al., 2021; Sallaberry et al., 2021). However, the relationship between protein-modified folding molecular networks and AD pathogenesis is complex and multifaceted (Rutledge et al., 2022). It involves various cellular and molecular mechanisms, including protein misfolding, aggregation, and clearance pathways, as well as neuroinflammation and oxidative stress (Crestini et al., 2022).

In 2019, the study conducted by Zhu investigated the differential expression of genes in GSE1297 and found that 16 genes were up-regulated and 14 genes were down-regulated significantly, as analyzed using *t*-test with a significance level of *p* < 0.05 (Guiqiong et al., 2019). Among these differentially expressed genes, functional enrichment analysis revealed that BAG2, a gene directly involved in the phosphorylation of Tau protein, was selected as a starting point for further exploration of the exact mechanism behind the occurrence and progression of AD. BAG2, as a nucleotide exchange factor (NEF) and co-chaperone protein of HSC70, regulates the folding efficiency of Tau protein (MAPT) (Arndt et al., 2005). HSC70 is involved in the recognition and binding of misfolded proteins, and BAG2 enhances this process by promoting the transfer of misfolded proteins from HSC70 to other chaperone proteins (Stricher et al., 2013). BAG2 interacts with HSC70’s nucleotide binding domain (NBD) as an NEF to accelerate the frequency of conformational change by stimulating HSC70’s ATPase activity (Xu et al., 2008). STUB1, also known as CHIP, is an E3 ubiquitin ligase that recognizes and ubiquitinates misfolded proteins, targeting them for degradation by the proteasome (Ferreira et al., 2013). In the process of lysosome degradation mediated by STUB1, the abnormal Tau protein combined with HSC70 is ubiquitinated to a target protein containing multiple ubiquitin chains, which can be recognized by the proteasome and be refolded to the normal structure (Petrucelli, 2004). Moreover, BAG2 can interact with E2 enzymes, inhibiting STUB1 activity and affecting the STUB1-mediated proteasomal degradation pathway (Schönbühler et al., 2016).

This study focuses on the relationship among BAG2, HSC70, STUB1, and MAPT, and discusses their roles in the pathogenesis of AD. Information was extracted from microarray and analyzed to further elaborate the folding and degradation pathways of Tau in AD. Changes of four gene expression patterns in patients in different stages were analyzed to find out the deep mechanism of the occurrence and development of AD. The findings of this study provide a better understanding of the molecular mechanisms underlying the development and progression of AD and may offer new targets for therapeutic interventions.

## 2. Materials and methods

### 2.1. Study description

In brief, all feature gene data are derived from analyses of Affymetrix Microarray data on 9 healthy subjects and 22 Alzheimer’s patients with hippocampal autopsies. The data were generated by the University of Kentucky College of Medicine and revealed on the NCBI data set as GSE1297. GPL96 (HG-U133A) Affymetrix Human genome U133a Single array was used to extract the expression information of the GSE1297 data set. All of the subjects have been divided into four groups based on MMSE (Mini-mental State Examination) criteria which are “Control,” “Incipient AD,” “Moderate,” and “Severe.”

### 2.2. Data preprocessing

In this paper, this study used matrix $G_{con},G_{inc},G_{\mathrm{mod}},G_{sev}$ to represent the data depending on the AD severity of samples:


$$G_{con}=\left[\begin{array}{ccc} g_{11} & \cdots & g_{1n}\\ \vdots & \ddots & \vdots \\ g_{m1} & \cdots & g_{mn} \end{array}\right]={\left(g_{ij}\right)}_{mn}$$

where m=22283 is the number of genes; n=9  is the number of subjects in the control group;


$$G_{inc}=\left[\begin{array}{ccc} g_{11} & \cdots & g_{1n}\\ \vdots & \ddots & \vdots \\ g_{m1} & \cdots & g_{mn} \end{array}\right]={\left(g_{ij}\right)}_{mn}$$

where m=22283 is the number of genes; n=7  is the number of subjects in the incipient group;


$$G_{\mathrm{mod}}=\left[\begin{array}{ccc} g_{11} & \cdots & g_{1n}\\ \vdots & \ddots & \vdots \\ g_{m1} & \cdots & g_{mn} \end{array}\right]={\left(g_{ij}\right)}_{mn}$$

where m=22283 is the number of genes; n=8  is the number of subjects in the moderate group;


$$G_{sev}=\left[\begin{array}{ccc} g_{11} & \cdots & g_{1n}\\ \vdots & \ddots & \vdots \\ g_{m1} & \cdots & g_{mn} \end{array}\right]={\left(g_{ij}\right)}_{mn}$$

where m=22283 is the number of genes; n=7  is the number of subjects in the severe group;

$g_{ij}$ represents the microarray expression value of the m-th gene in the n-th sample. Generally speaking, each row of matrix G can be seen as one gene and each column of matrix G can be seen as one sample. The number of genes is much larger than that of the samples. Since microarray data were obtained in different experimental environments (Gilad and Borevitz, 2006), thus, log-transformation was used in matrix G to eliminate the magnitude difference of data and enable different samples to be compared. The details of matrix $X_{con}$ construction are as follows:


(1) $$x_{ij}=\log_{10}\left(g_{ij}+1\right)\#$$

$$\begin{array}{c} \;X_{con}=\left[\begin{array}{ccc} x_{11} & \cdots & x_{1n}\\ \vdots & \ddots & \vdots \\ x_{m1} & \cdots & x_{mn} \end{array}\right]={\left(x_{ij}\right)}_{mn}\# \end{array}$$

where m=22283 is the number of genes; n=9  is the number of subjects in the control group; each row m=1,…,22283 represents the gene expression value; column n=1,…,9 represents an individual, and the vector $x_{ij}$ is the expression value after the log-transformation of original microarray data. The purpose of log transformation is to ensure that the distribution of the expression data is consistent with a normal distribution. It is crucial for statistical analysis since most of them require a normally distributed sample (Curran-Everett, 2018). Then, $X_{con}$ denotes the matrix of nine control samples, $X_{inc}$ denotes the matrix of seven incipient patients, $X_{\mathrm{mod}}$ denotes the matrix of eight moderate patients and $X_{sev}$ denotes the matrix of seven severe patients.

### 2.3. Correlation coefficient matrix decomposition analysis

In this section, we extract the expression values of s genes from $X_{con}$, $X_{inc}$, $X_{\mathrm{mod}}$ and $X_{sev}$ and process the inner product between them, represented as matrices $R_{con,}R_{inc},R_{\mathrm{mod}},R_{sev}$ consisting of vector ${\vec{a}}_{i},{\vec{b}}_{i},{\vec{c}}_{i}\;and\;{\vec{d}}_{i}$ respectively. Below is the example of establishing $R_{con}$:


$${\vec{a}}_{i}=\left(x_{i1},\ldots ,x_{in}\right)$$

$${\vec{a}}_{i}•{\vec{a}}_{j}=x_{i1}x_{j1}+x_{i2}x_{j2}+\ldots +x_{in}x_{jn}$$

(2) $$\begin{array}{c} R_{con}=\left[\begin{array}{c} {\vec{a}}_{1}\\ \vdots \\ {\vec{a}}_{s} \end{array}\right]{\left[\begin{array}{c} {\vec{a}}_{1}\\ \vdots \\ {\vec{a}}_{s} \end{array}\right]}^{T}=\left[\begin{array}{ccc} {\vec{a}}_{1}•{\vec{a}}_{1} & \cdots & {\vec{a}}_{1}•{\vec{a}}_{s}\\ \vdots & \ddots & \vdots \\ {\vec{a}}_{s}•{\vec{a}}_{1} & \cdots & {\vec{a}}_{s}•{\vec{a}}_{s} \end{array}\right]\# \end{array}$$

Here, this study focuses on the genes with protein folding and degradation functions. Therefore, s is a part of $X_{con},i$ and j represent the i-th and j-th gene in the geneset s. n is the number of samples in the control group which is 9. $R_{con}\;$is the matrix with rows corresponding to gene expression values, representing the dot product between s genes.

In this situation, $R_{con}$ is a symmetric and positive semi-definite matrix. Thus, matrix $R_{con}$ should contain correlation information between s genes in normal subjects. This kind of information should vary significantly in matrix $R_{sev}$, reflected by the eigenvalue change. In this analogy, we can construct $R_{inc},R_{\mathrm{mod}},R_{sev}$ while n is 7, 8, 7, respectively.

Moreover, valuable information such as the correlation between genes and disease is hidden in matrix R. However, it is challenging to find it since one matrix may include tens of thousands of elements. The matrix can process eigenvalue decomposition using formula (3) to extract valuable information:


(3) $$\begin{array}{c} R_{\mathrm{con}}=Q\left[\begin{array}{ccc} \lambda_{1} & & \\ & \ddots & \\ & & \lambda_{i} \end{array}\right]Q^{-1}\# \end{array}$$

Where Q is the eigenvector matrix and λ is the matrix consisting of eigenvalues of semi-definite matrix $R_{con},$ the i-th of matrix Q is the eigenvector of the $\lambda_{i}$. $\lambda =\left\{\lambda_{1},\ldots ,\lambda_{s}\right\}$ and $Q^{-1}=Q^{T}$. Moreover, It is essential for the matrix Q is invertible, it guarantees eigenvalue decomposition does not affect gene correlations. By convention, λ should be in order $\lambda_{1}\geq \lambda_{2}\geq \ldots \geq \lambda_{\begin{array}{c} s \end{array}}\;$and transforms into percentage used formula λi=λi∑k=1sλk. So, the value of $\lambda_{i}$ should be between 0 and 1, the sum of them equals 1, the first of λ should be greater than all λ.

The eigenvectors and eigenvalues of the inner product matrix can reveal important information about the underlying structure of the gene expression data. In this case, the eigenvectors represent the directions of maximum variation in the data, while the corresponding eigenvalues represent the magnitude of that variation. The eigenvalues represent the matrix’s intrinsic characteristics and directly indicate the correlation between genes. For example, if we treat the matrix as a motion, then the eigenvalue is the velocity of the motion, the eigenvector is the direction of the motion.

### 2.4. WGCNA analysis

To reveal the *MAPT* folding network correlated with AD, analysis of gene co-expression was performed via this weighted gene co-expression network analysis (WGCNA). It is an algorithm for mining module information from microarray data. This method defines the module as a group of genes with similar expression profiles. To identify modules, WGCNA constructs a gene co-expression network where nodes represent genes and edges represent the strength of the correlation between genes. The network is then clustered into modules based on the topological overlap measure, which captures both the strength and the distribution of the connections between genes. If some genes always have similar expression changes in a physiological process, it is reasonable to believe that these genes are functionally related and can be defined as a module (Langfelder and Horvath, 2008).

First, this study used all logarithmic transformation data to test its availability, and used “WGCNA” R package to construct a gene co-expression network. Subsequently, all genes need to be calculated with Spearman Correlation Coefficient for any two of them. The formula for the Correlation Coefficient is as follows:


(4) $$\begin{array}{c} s_{ij}={\left|corr\left({\vec{x}}_{i},{\vec{x}}_{j}\right)\right|}^{\beta }\# \end{array}$$

In this formula, $corr\left({\vec{x}}_{i},{\vec{x}}_{j}\right)$ is the Spearman correlatoin coefficient between gene i and j, i and j represent two different genes, ${\vec{x}}_{i}$ and ${\vec{x}}_{j}$ are their expression value. β is the soft power of the correlation coefficient, and the strength of correlation can be changed by adjusting it. After that, this study constructed a co-expression module by clustering genes based on the $s_{ij}$ using the average linkage hierarchical clustering method and merge cut height supposed to be set for merging branches below this cut height. The genes in the same module should have a high $s_{ij}$ value, and different modules should have no significant relationship.

### 2.5. Identifying different expressive genes

Genes need to be identified as differentially expressed up-regulated or down-regulated genes by independent *t*-testing with a confidence interval of 0.05. The differentially expressed genes were clustered by biological function. For an exciting cluster, this study used Python and formula 3 with eigenvalue decomposition to a matrix consisting of $R_{con},R_{inc},R_{\mathrm{mod}}$, $R_{sev}$ respectively. For the 4 group patients, the changing rate of eigenvalues for different matrices indicates how the relationship between them change with the degree of the disease. If the eigenvalues are approximately 0, it indicates that there is at least one gene regulated by other genes in AD patients.

### 2.6. Neural network predict MMSE

Mini-Mental State Examination (MMSE) is a brief screening test that is quantitatively used to assess people’s cognitive capabilities conducted by experienced physicians, in which subjective and empirical issues exist, so it is valuable to use artificial intelligence to objectively evaluate mental state (Paul et al., 2022). Neural Network (NN) is a popular method to regression any function based on the sum of unlimited different Sigmoid function (Kriegeskorte and Golan, 2019). In this article, this study constructed a neural network to predict MMSE from *BAG2, HSC70, STUB1*, and *MAPT* expression values. It includes one input layer consisting of 4 nodes, one hidden layer consisting of 32 nodes, and one output layer consisting of 1 node. In particular, neurons or nodes represent different parameters, and layers represent a column of parameters (SiTaula et al., 2021).


(5) $$\begin{array}{c} y=b+{\vec{c}}^{T}\vec{a}\# \end{array}$$

$$\;\vec{a}=\left(a_{1},\ldots ,a_{32}\right)$$

(6) $$\begin{array}{c} a_{i}=Sigmoid\left(v_{i}\right)=\frac{1}{1+e^{-v_{i}}};i=\left(1,\ldots ,32\right)\# \end{array}$$

(7) $$\begin{array}{c} \vec{v}=\vec{k}+W\vec{x}=\left(v_{1},\ldots ,v_{32}\right)\# \end{array}$$

In formula 5 ~ 7, y is the value of MMSE, is the output of NN; $\vec{x}\;$is a 4 dimensions input vector and supports being normalized; b is the bias value of the output layer and $\vec{k}$ is the 32 dimensions bias vector of the hidden layer; W is a 32 × 4 parameter matrix of the output layer and $\vec{c}$ is 32 dimensions parameter vector of the hidden layer; $\vec{a}$ represents the 32 hidden nodes of the model. Formula 7 represents the transformation from the input layer to the hidden layer, a Sigmoid function represented by formula 6. In this NN, the parameters should be adjusted by optimization techniques Stochastic Gradient Descent (SGD) base Mean Square Error (MSE). These techniques are efficient and have low computational complexity in NN, which have found wide application in Machining Learning (Lei et al., 2020).

### 2.7. Support vectors machines classification

Support vector machines (SVM) can provide more efficacious classification performances than other machine learning techniques in biology gene data. The fundamental theory is mapping origin data into a higher dimension and classifying it by a plane called Hyperplane. For example, if data have n dimensions and are unable to be classified by a hyperplane, then this data should be mapped into n + 1 dimensions using Radial Basis Function (RBF). RBF can approximate multivariable functions by linear combinations of terms based on a single univariate function. It usually acts as a mapping function in SVM (Buhmann, 2010). The detail of the SVM model can be defined as follows:


$$X=\left[\begin{array}{c} {\vec{x}}_{1}\\ \vdots \\ {\vec{x}}_{s} \end{array}\right]$$

$${\vec{x}}_{i}=\left(x_{i1},x_{i2},\ldots ,x_{ij}\right);i=\left(1,\ldots ,s\right)$$

$$Y=\left[\begin{array}{c} {\vec{y}}_{1}\\ \vdots \\ {\vec{y}}_{s} \end{array}\right]$$

(8) $$\begin{array}{c} {\vec{y}}_{1}=\left(y_{i1},y_{i2},\ldots ,y_{ij},y_{i\left(j+1\right)}\right);i=\left(1,\ldots ,s\right)\# \end{array}$$

(9) $$\begin{array}{c} y_{ij}=similarity\left({\vec{x}}_{i},{\vec{l}}_{j}\right)=e^{-\frac{{\vec{x}}_{i}-{\vec{l}}_{j}{}^{2}}{2\sigma^{2}}};i=\left(1,\ldots ,s\right)\# \end{array}$$

where ${\vec{x}}_{i}$ is the i-th gene expression value in matrix X, s is the number of genes used in the SVM model, j is the number of samples; Y is the input of the model, mapped by ${\vec{x}}_{i}$ into $\left(j+1\right)$ dimensions using RBF function. Formula 9 is the detail of the RBF function, $\vec{l}$ is the j dimensions vector and there are $\left(j+1\right)$ vectors l in the model, ${\vec{l}}_{j}$ is the j-th vector of $\vec{l}$. $\sigma^{2}$ is the hyper-parameter of the model, usually chosen as 0.5.


(10) $$\begin{array}{c} WY+B=0\# \end{array}$$

Where W is the weight matrix, Y is the input feature matrix and B is the bias matrix. In this article, this study trained the SVM model to find a W and B to maximize the margin $1/W^{2}$. To evaluate SVM performance, this study used Sensitivity and Specificity to establish Receiver Operating Characteristics (ROC). The details are as follows:


(11) $$\begin{array}{c} y=\frac{p}{p+n}\# \end{array}$$

(12) $$\begin{array}{c} z=\frac{t}{f+t}\# \end{array}$$

In formulas 11 and 12, y is the value of sensitivity, z is the value of specificity, p means True Positive which is correctly classified as positive samples; n means False Negative represents the number of samples incorrectly classified as positive; t means True Negative corresponding to the number of negative samples correctly classified; f means False Positive, is the number of negative samples incorrectly classified.

## 3. Results

### 3.1. Matrix decomposition

The eigenvectors and eigenvalues of the inner product matrix can provide crucial information about the underlying structure of gene expression data. In this section, we employed matrix decomposition analysis to gain deeper insights into the molecular mechanisms underlying disease progression. Specifically, as shown in Supplementary Tables S1–S4, we extracted the expression values of four genes (BAG2, HSC70, STUB1, and MAPT) and computed the inner product matrix using Formula 2. We then analyzed the resulting inner product matrix using matrix decomposition to evaluate the four groups.

**Figure 1:**
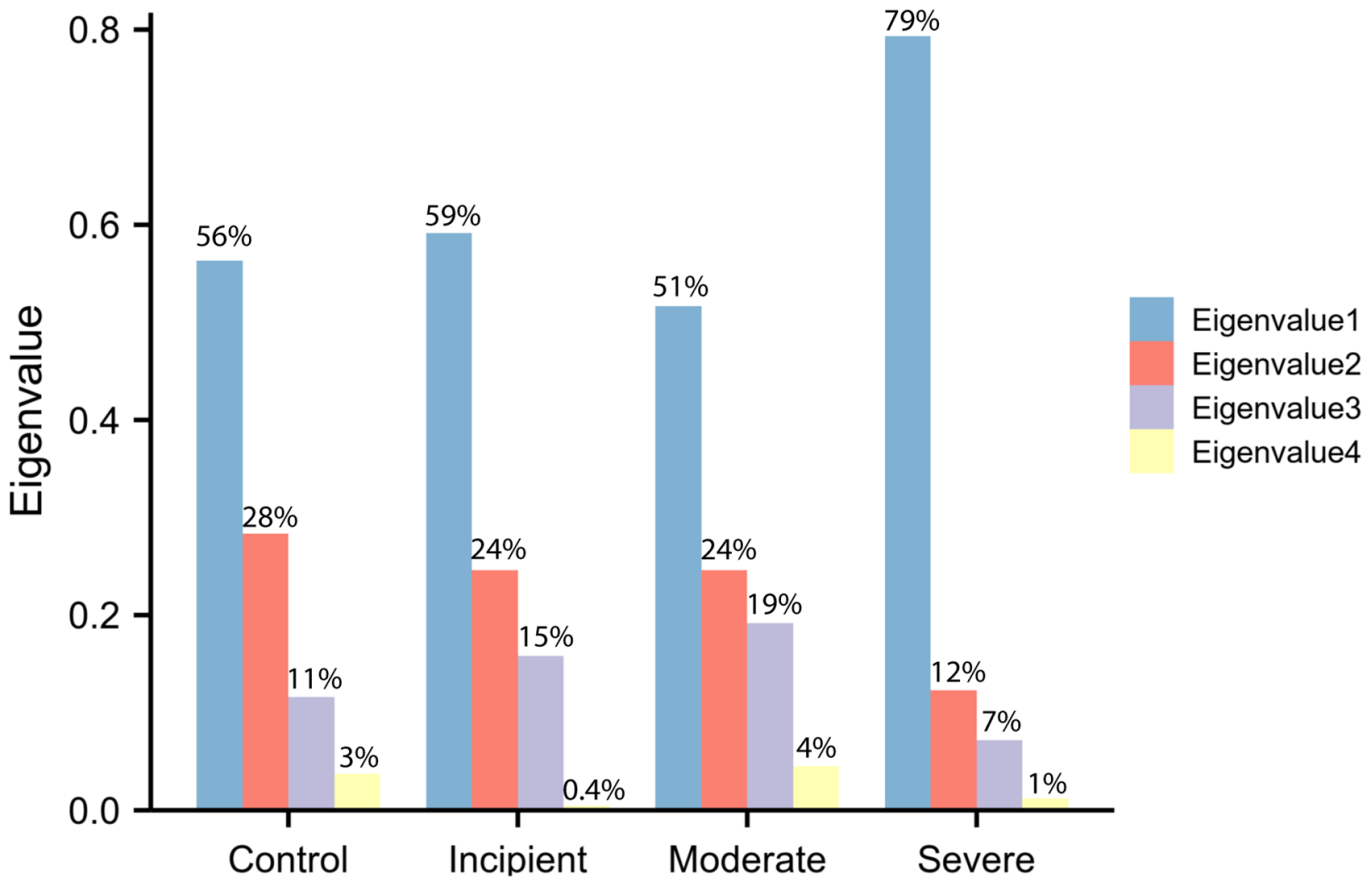
Eigenvalues of the expression matrix. Eigenvalues were computed on the basis of the inner product matrix consisting of BAG2, STUB1, HSC70, MAPT (Tau). The *x*-axis represents the level of the group and the *y*-axis represents the eigenvalues that have been percentage transformed, thus the sum of eigenvalues equals 1. The cyan bar is the maximum eigenvalue and the light yellow bar is the minimum eigenvalue. The biggest eigenvalues in the control group are 0.56 and in the severe group are 0.79, which indicates that the correlation among genes is significantly modified during the development of disease.

Our findings of eigenvalues, depicted in Figure 1, suggest that the difference between eigenvalues changed less in the first three phases of the disease, whereas the divergence of distribution became more skewed toward the extremes in the severe stage. This suggests that the overall correlation among these four genes decreases as the condition worsens, resulting in reduced efficiency of MAPT folding and degradation. The maximum eigenvalues increased from approximately 0.56 to around 0.8, indicating that at least one of the four genes plays a major and triggering role in the folding and degradation function when the disease becomes more severe. Moreover, while eigenvalues represent the magnitude of that variation, the eigenvectors reveal the direction of the variation.

The eigenvectors corresponding to the eigenvalues in the control group are presented in Table 1, while Table 2 shows the eigenvectors of the severe group. The first column of the eigenvectors matrix corresponds to the largest eigenvalue, which is the most important element. These results suggest that the underlying gene expression patterns have changed between the two groups. For example, the eigenvector of the biggest eigenvalue in the control group is [0.16, −0.57, 0.51, 0.62], which contributes 56% of the information in the matrix. However, in the severe group, not only did the eigenvalue change significantly, but also the sign of the elements in the eigenvector reversed. This change in sign for all elements indicates a shift in the direction of maximum variation in the gene expression data.

**Table 1:** The eigenvector of inner product in control group.

	$\lambda_{1}$	$\lambda_{2}$	$\lambda_{3}$	$\lambda_{4}$
	0.1603998	−0.86496189	0.43869841	0.18345718
	−0.57748234	0.26959268	0.49608849	0.58968648
	0.50539008	0.39466183	0.71249652	−0.28490634
	0.62077928	0.15297958	−0.23192376	0.73310415

**Table 2:** The eigenvector of inner product in severe group.

	$\lambda_{1}$	$\lambda_{2}$	$\lambda_{3}$	$\lambda_{4}$
	−0.47158612	0.4301255	0.75178616	0.16557825
	0.47986946	−0.34906073	0.61507755	−0.51919314
	−0.49541599	0.15885334	−0.2199049	−0.82520934
	−0.54944958	−0.81726022	0.09021649	0.14849873

**Figure 2:**
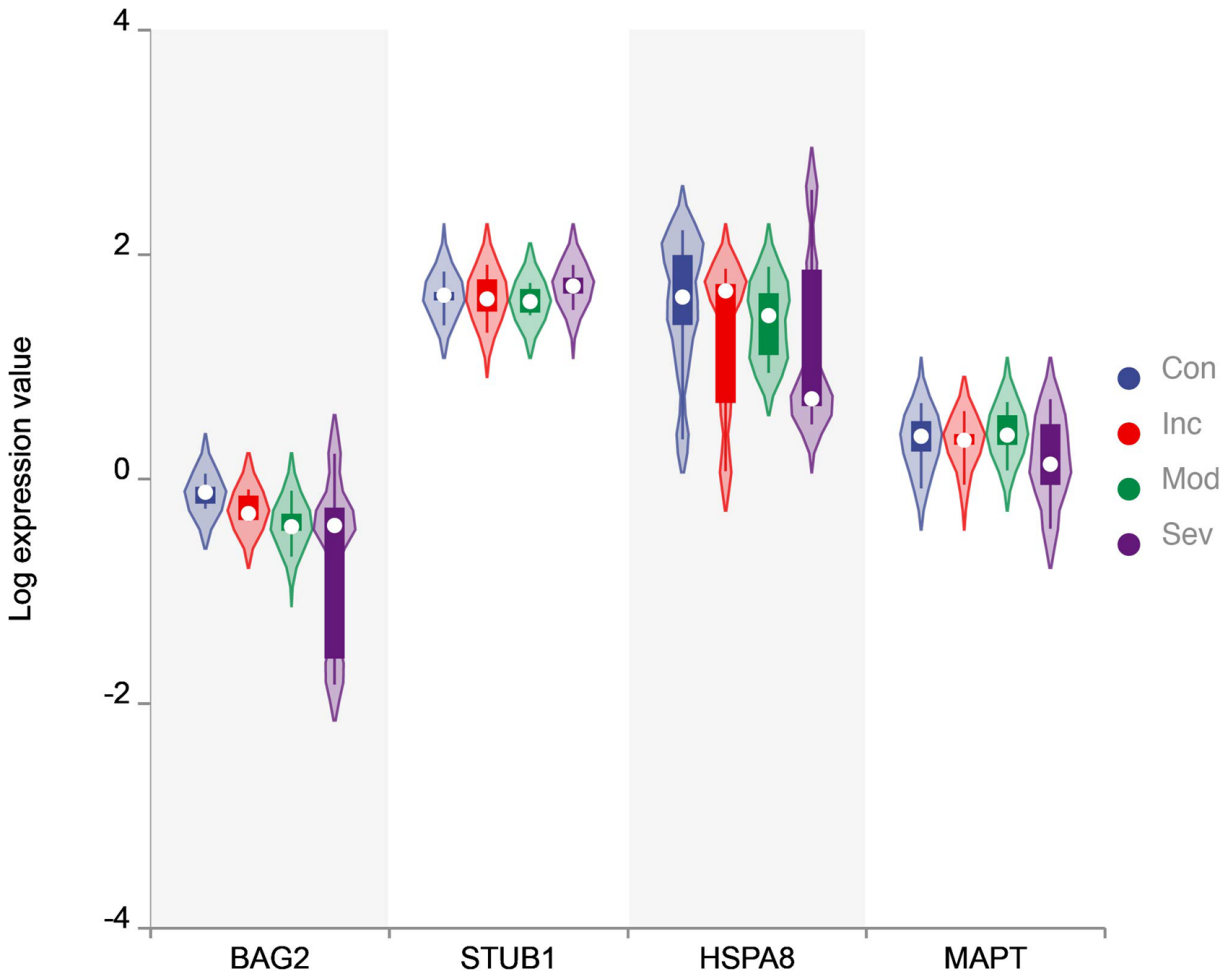
The violin plot of expression value. Violin plot of BAG2, STUB1, HSC70, and MAPT levels in four groups. The *y*-axis is the gene value after Log transformation. The plot displays the distribution of expression values for each group as a density curve, with thicker sections indicating a higher density of data points. The median expression level is represented by a white circycle, and the interquartile range (IQR) is indicated by a box. The width of the violins reflects the density of data points at each expression level. BAG2 is one of the significantly differential genes evaluated by two-sample *t*-test.

Furthermore, the eigenvector with the largest eigenvalue represents the direction of maximum variability in the data. In the control group, the genes BAG2, STUB1, HSC70, and MAPT had positive contributions to this direction of maximum variation, while in the severe group, the same genes had negative contributions. This suggests that the coordination of gene expression patterns has shifted from a positive to a negative correlation between these genes in the severe group compared to the control group. Notably, BAG2 may act as a triggering protein since its expression consistently and significantly declines while the other proteins remain unchanged, as demonstrated in Figure 2. Overall, the value of eigenvalues and the signs of eigenvectors suggest that the coordination within the molecular network consisting of BAG2, HSC70, STUB1, and MAPT changed significantly.

### 3.2. WGCNA network construction and identification of genes

**Figure 3:**
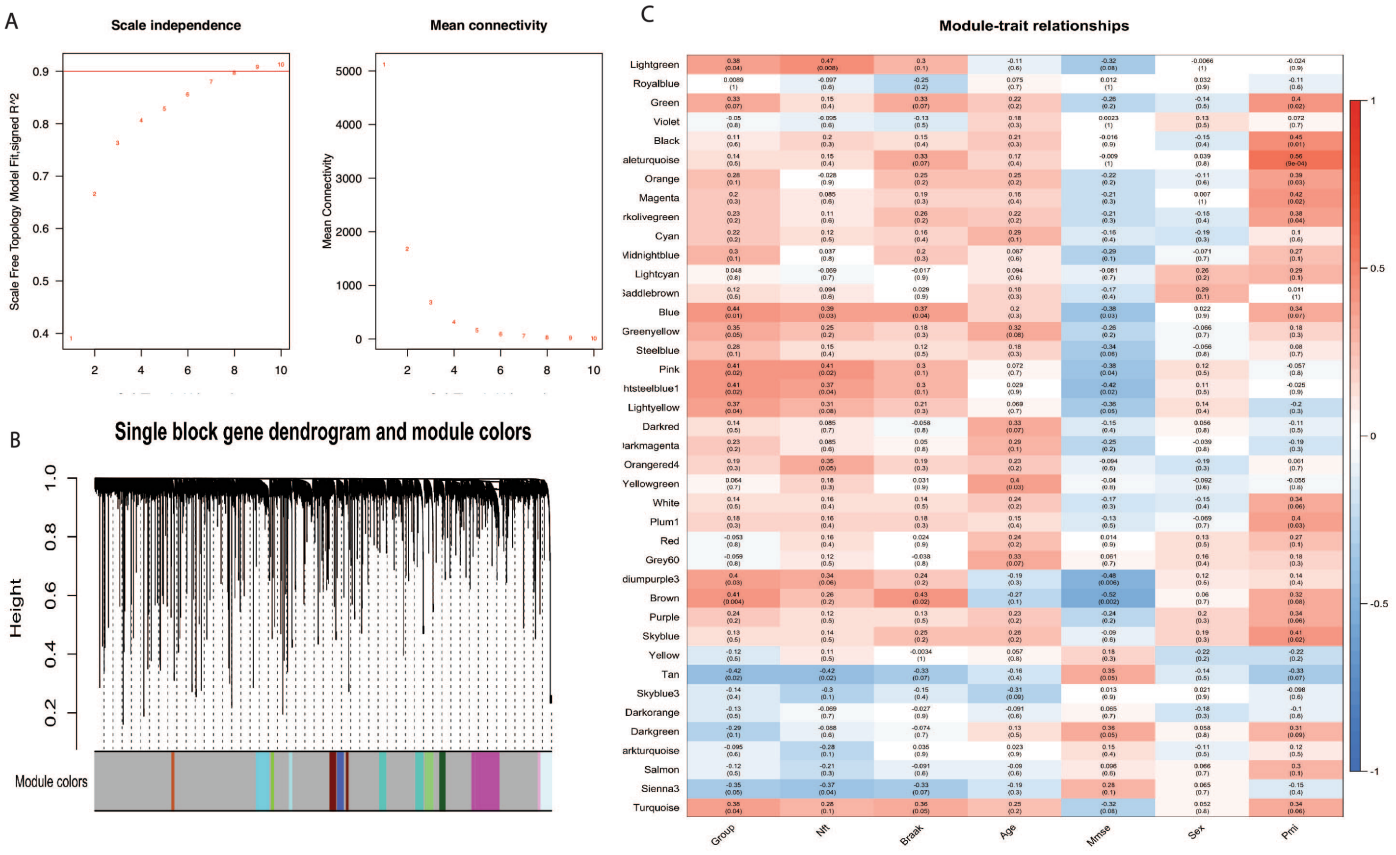
WGCNA analysis in Alzheimer disease. BAG2, STUB1, and MAPT belong to module blue, HSC70 belongs to module yellow. **(A)** The influence of various soft threshold powers on module independence and mean connectivity. **(B)** Dendrogram of all expressed genes clustered based on the soft power eight. The color band shows the result obtained from the single-block analysis. **(C)** The module-trait relationship heatmap of the correlation between the seven clinical traits and module eigengenes. Each row corresponds to a module and column to a feature; 20 modules were selected from all 41 modules for a pithy view.

We performed a Weighted Gene Co-expression Network Analysis (WGCNA) to investigate the expression patterns and coordination of BAG2, STUB1, HSC70, and MAPT in Alzheimer’s disease (AD) patients. WGCNA is a method that identifies co-expressed genes across different samples or conditions and groups them into modules. Our analysis involved extracting clinical attributes such as Group-NFT-BRAAK-AGE-MMSE-SEX-PMI directly from the original data, excluding any irrelevant data, and transforming the sample groups into numerical values (control group = 1, severe group = 4). We also log-transformed the gene expression data and established a scale-free co-expression network. To ensure module independence, we applied a soft threshold β of 8 and set the merge cut height to 0.25 (Figure 3A). We used formula 4 to perform hierarchical clustering of genes and obtain a hierarchical clustering tree. Using the dynamic tree cutting method, we defined the minimum number of genes per module as 30 and selected intermediate level classification to identify key clusters. Next, we classified the genes that were not assigned to any cluster into different clusters based on relevance, resulting in a total of 41 modules (Figure 3B). The genes that could not be classified into any modules were grouped together into the Grey module.

Figure 3C represents the relationships between modules and traits. We removed the grey module from the analysis, leaving 40 modules identified by WGCNA. Using the ‘color’ attribute of the WGCNA network in R, we determined the module membership of specific genes. Our analysis showed that BAG2, STUB1, and MAPT belonged to module blue, while HSC70 belonged to module yellow, indicating that these four genes have similar expression patterns and are functionally related. The results shown in Figure 3C also indicate that module blue, which consists of three out of the four genes, exhibited the highest correlation with disease severity (control vs. severe) among all the identified modules with a value of *p* of 0.01, suggesting a significant association between the co-expression pattern of genes in module blue and the disease condition. The finding of WGCNA indicates that BAG2, STUB1, HSC70, and MAPT have similar expression patterns and play important roles in the pathogenesis of the disease.

### 3.3. Neural network predict MMSE

**Figure 4:**
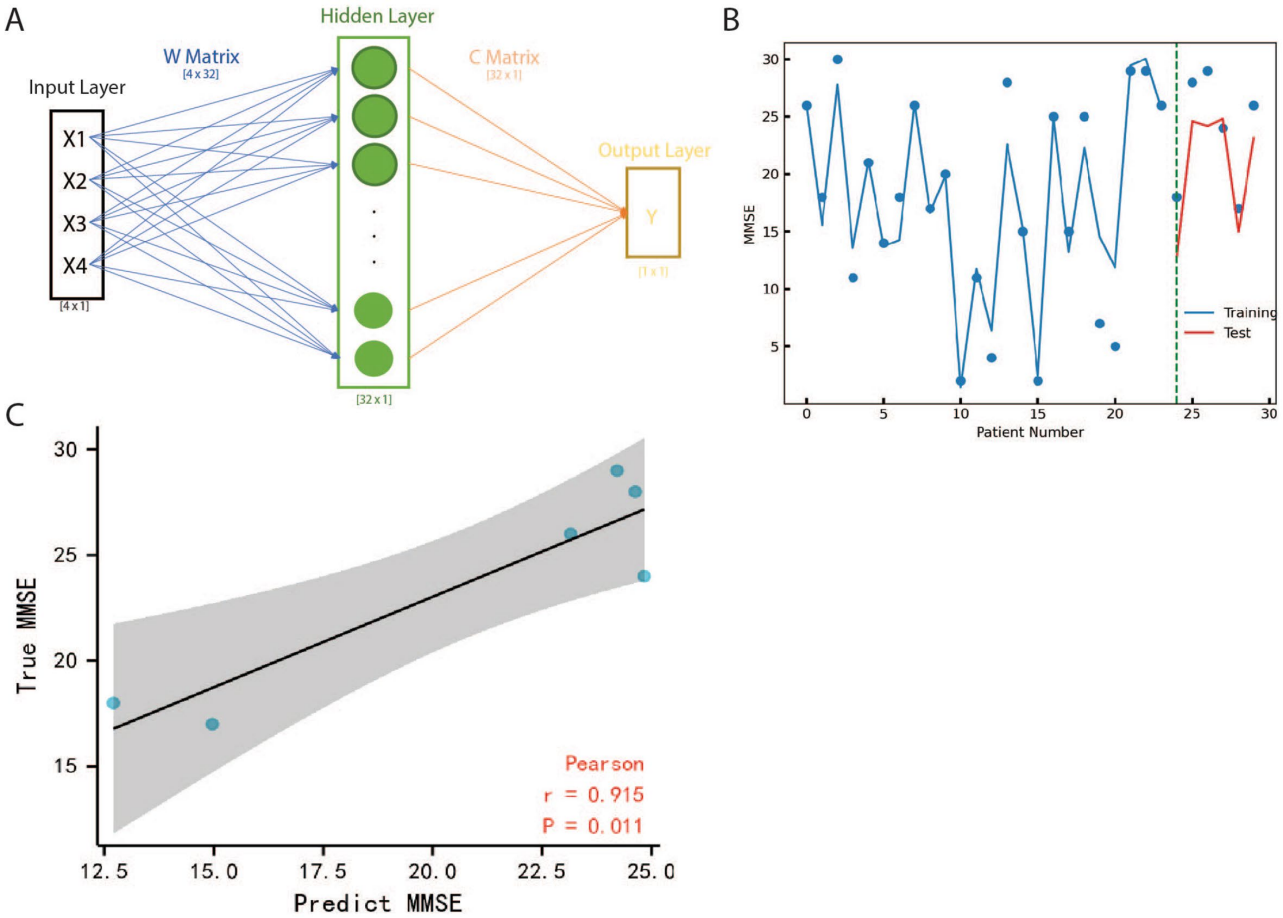
The structure and results of Neural Network. **(A)** The input layer is a four dimensions vector that represents the expression value of BAG2, HSC70, STUB1 and MAPT. Y is the predicted MMSE value by NN. Matrix W and Care weight parameters composed of relative information between input and output. **(B)** Curve fitting based on Neural Network with MMSE scatters on Test and Training Data. *X* indicates different patients, *Y* is the value of MMSE, the blue line represents the fitting result of training data, and the red line represents the fitting result of test data. The green dash line is the boundary between training and testing patients. **(C)** Pearson coefficient analysis of Neural Network result, the x-axis is the MMSE and the *y*-axis is the predicted MMSE for test patients, the black line is the fitting line and the grey plane represents the 95% confidence interval. The correlation coefficient is represented as *r* which is 0.915 showing the results of NN are close to true value. All the data points scatter in the grey plane and the value of *p* of the hypothesis test is only 0.011, which means our model is robust as well as highly accurate.

In this study, a three-layer neural network was constructed using Python 3.8 with Torch package 2.6 to predict MMSE value of patients based on the expression value of BAG2, HSC70, STUB1, and MAPT. The architecture of the neural network is presented in Figure 4A, and the study divided the GSE1297 datasets into two subsets, namely training and testing datasets, to ensure the model’s generalization ability to new and unseen data. A separate testing dataset was utilized to evaluate the neural network model’s performance on data that had not been used during the training phase to prevent overfitting, a common issue in machine learning models (Wei and Dunbrack, 2013). Supplementary Tables S5, S6 provide the details of the data and the outcome of the neural network. To estimate the neural network model, Spearman’s correlations analysis with a confidence interval set at 95% was utilized. Figure 4B illustrates that each point represents a different sample and the line represents the neural network model, while the green dash line splits the test and training data. Additionally, Figure 4C demonstrates that the predicted MMSE has a strong correlation (*r* = 0.915) with the true MMSE, and this kind of relationship is highly credible (*p* = 0.011).

This model’s importance lies in its ability to accurately predict patient MMSE values based on the expression of specific genes, namely BAG2, HSC70, STUB1, and MAPT. The model has demonstrated a strong correlation between predicted and actual MMSE values, indicating its reliability and validity. By using this neural network model, it is possible to obtain valuable insights into a patient’s mental status without the need for time-consuming tests, which can be particularly important in clinical settings. Additionally, the model reveals a strong relationship between these genes and AD, providing new avenues for further research into the underlying mechanisms of the disease.

### 3.4. Support vectors machines classification

**Figure 5:**
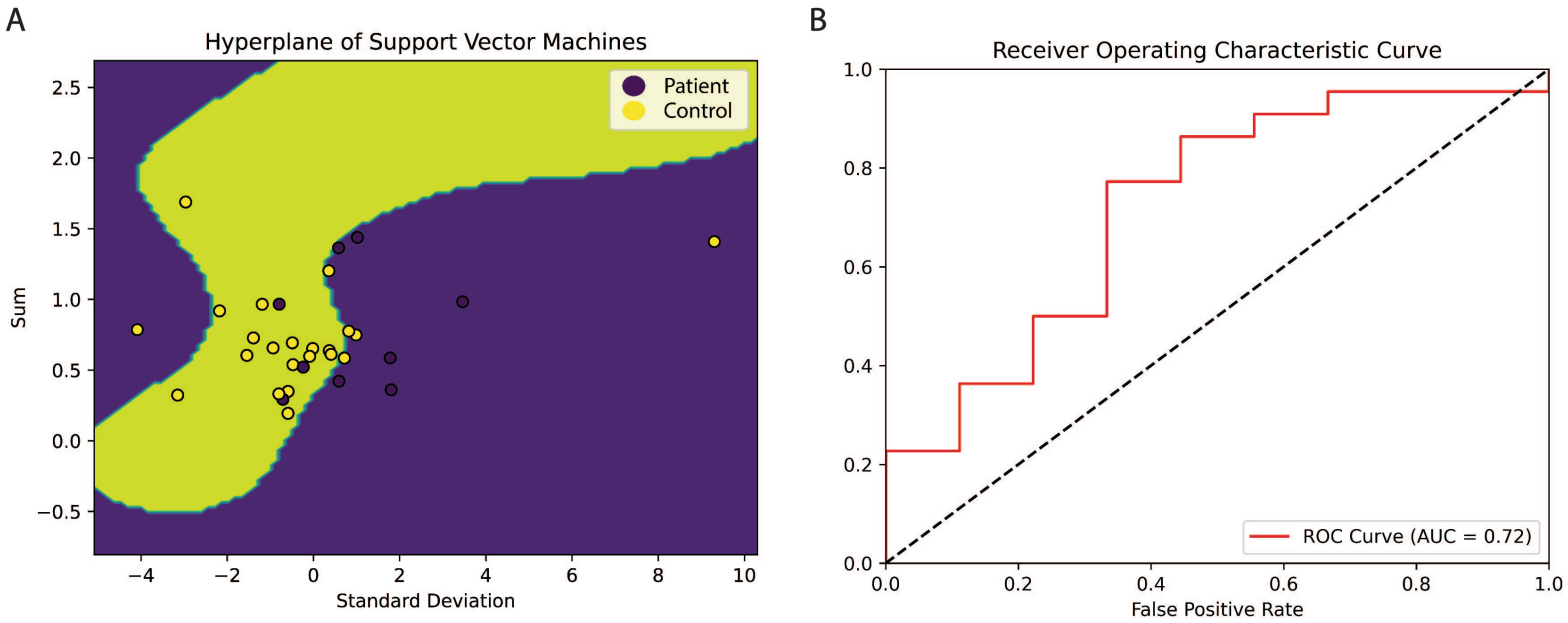
The hyperplane and ROC cure of SVM. **(A)** The hyperplane of SVM. This study used the value of sum and standard deviation as X1 and X2 to illustrate better that a yellow background characterizes the location and shape of the hyperplane. Two dimensions input vectors have been mapped into three-dimension, then classified by a hyperplane. The SVM was trained by 361 samples from GSE1522 and verified with 31 pieces in GSE1297. The result shows that a hyperplane can classify most samples, it suggests that these four genes may play a decisive role in the formation of AD. **(B)** The cure of Receiver Operating Characteristic. *X* is the false positive rate equal (1-specificity), and *Y* is the recall term of sensitivity. The area below the red line is the accuracy of SVM, which is 0.72.

In this section, this study obtained 180 samples in the control group and 181 samples in the patient’s group from the GSE15222 project of Alzheimer International Institution as SVM training data and 31 samples from GSE1297 as verifying data to make up for the few shortcomings of the training samples in NN. The sum and standard deviation of expression values of the four genes were used as inputs for SVM, which transformed the two-dimensional input vector into a three-dimensional space using RBF. As depicted in Figure 5A, the majority of samples were classified by a hyperplane, indicating the effectiveness of the model in distinguishing between control and patient groups.

To further evaluate the performance of the model, a ROC curve was plotted based on the sensitivity and 1-specificity values. The dashed line in Figure 5B represents the average line, indicating that the positive rate exceeds the negative rate if a point falls beyond it. The area under the ROC curve was calculated to be 0.72, indicating a high level of accuracy in identifying Alzheimer’s disease patients based on the expression levels of the four genes.

## 4. Discussion

It is widely known that Beta-amyloid and Tau proetin are related to Alzheimer’s disease pathology and not directly related to the protein folding system. However, there are molecular markers related to the protein folding system that are used in research to study protein folding and misfolding (Sallaberry et al., 2021). Examples of such markers include chaperone proteins, which help to maintain proper protein folding, and molecular chaperones, which recognize misfolded proteins and target them for degradation. Other examples include post-translational modifications such as ubiquitination and phosphorylation, which can affect protein folding and stability (Stocker et al., 2021). Although molecular markers related to protein folding may not be used for diagnosing or understanding Alzheimer’s disease specifically, they are important in understanding the basic mechanisms of protein folding and how misfolding can lead to various diseases including neurodegenerative diseases. In this study, the alterations in the expression of four genes were examined in individuals at various stages of Alzheimer’s disease to elucidate the role of BAG2, HSC70, STUB1 in folding and degradation of Tau in disease pathogenesis and progression.

The study focused on analyzing the gene expression levels of Affymetrix Microarray, including 9 healthy subjects and 22 Alzheimer’s patients. In a previous study, team performed normalization and *t*-testing on the expression data of all 22,283 genes, resulting in the identification of 16 up-regulated genes and 14 down-regulated genes (Guiqiong et al., 2019). The differential expression genes were described in the Supplementary Tables S7, S8. Of note, BAG2 was found to be one of the down-regulated genes. Further analysis of the Uniport database of BAG2^1^ revealed a strong correlation with HSC70 and STUB1. Based on the literature survey and Uniport database findings, this article focuses on the effects on the protein folding and degradation system and Alzheimer’s disease when the molecular network consisting of BAG2, HSC70, STUB1 and MAPT is dysregulated.

As shown in Figure 1, the results of matrix decomposition analysis showed that the difference between eigenvalues changed less in the early stages of the disease, while the distribution became more skewed toward the extremes in the severe stage. This indicates that the overall correlation among these four genes decreases as the condition worsens, resulting in reduced efficiency of MAPT folding and degradation. Moreover, the eigenvectors revealed a shift in the direction of maximum variation in the gene expression data (Table 2), with BAG2 potentially acting as a triggering protein since its expression consistently and significantly declined while the other proteins remained unchanged (Figure 2). To further investigate the expression patterns and coordination of BAG2, STUB1, HSC70, and MAPT in Alzheimer’s disease patients, we performed a WGCNA analysis. Figure 3 showed that these four genes have similar expression patterns and are functionally related, with BAG2, STUB1, and MAPT belonging to module blue, while HSC70 belonged to module yellow. The module blue exhibited the highest correlation with disease severity among all the identified modules, suggesting a significant association between the co-expression pattern of genes in module blue and the disease condition.

The artificial intelligence models were used to calculate the mapping relationship between the expression levels of the four hub genes and clinical parameters: the results of NN (Figure 4) showed that there was a significant linear relationship between the expression levels of the four core genes and the golden index for AD diagnosis, MMSE (Spearman The correlation coefficient is 0.97). The SVM model trained by GSE15222 and tested by GSE1297 (Figure 5), showed that the expression levels of the four hub genes could be used to accurately predict the MMSE score of the subjects, and the subjects were classified to determine whether they suffer from AD (The area under the ROC curve is 0.72). We trained two reliable artificial intelligence models to predict the status of AD subjects at different stages. The results showed that the expression patterns of four hub genes (BAG2, HSC70, STUB1, and MAPT) affecting protein folding and degradation were significantly correlated with clinical diagnostic indicators at all stages of AD progression.

**Figure 6:**
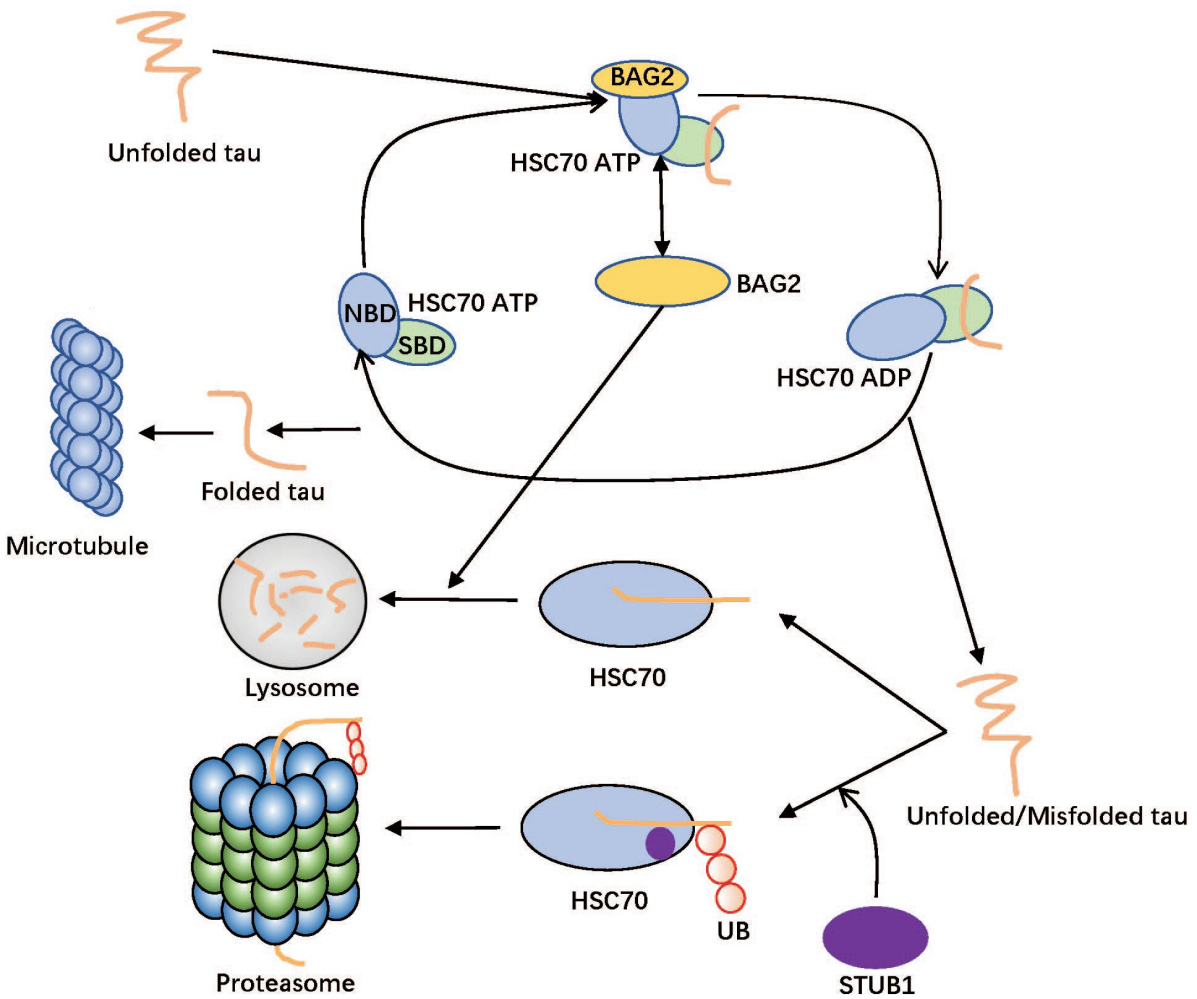
Diagram for the folding and degradation system of Tau. The HSC70 is divided into two domains in this figure: nucleotide-binding domain (NBD) and substrate-binding domain (SBD). Unfolded/Misfolded tau can be folded in the SBD of ADP form of HSC70 and degraded in lysosome or proteasome in cooperation with BAG2 and STUB1, respectively. In proteasome pathway, the ubiquitin ligase STUB1 catalyzes transfer of ubiquitin from an E2 enzyme to form a covalent bond with tau. Therefore, tau supports being tagged with ubiquitin before the lysosome degradation process with STUB1. BAG2 as Nucleotide exchange factor (NEF) of HSC70 binding to NBD plays a dual role to accelerate folding efficiency and assist tau in delivering tau to the lysosome.

Figure 6 shows a brief interaction among the hub genes. As a molecular chaperone, HSC70 corrects the misfolding of nascent peptides and promotes the formation of the correct structure of the target protein with biological functions (such as natural Tau) (Lo et al., 2004). HSC70 consists of two main domains: the N-terminal nucleotide-binding domain (NBD) and the C-terminal substrate-binding domain (SBD). The NBD is responsible for binding and hydrolyzing ATP, which drives the conformational changes required for substrate binding and release (Schuermann et al., 2008). The SBD is responsible for binding to unfolded or misfolded proteins and facilitating their folding or targeting them for degradation (Khachatoorian et al., 2014). As a common partner of HSC70, BAG2 can play a crucial role in the degradation and folding of Tau by affecting the proteasome and lysosomal systems (Schönbühler et al., 2016), and BAG2 can also interact with E2 enzymes, thereby inhibiting STUB1 activity and affects the STUB1-mediated proteasomal degradation pathway (Quintana-Gallardo et al., 2019). There are two roles of HSC70 in AD development. On the one hand, HSC70 can recruit local Tau in the form of ATP binding and fold them in the form of ADP binding in the substrate binding domain (SBD) (Papsdorf et al., 2019). BAG2 in the surrounding environment can combine with HSC70 (NBD) as a nucleotide exchange factor (NEF) to accelerate the frequency of conformational change by stimulating HSC70 ATPase activity (Bracher and Verghese, 2015). On the other hand, HSC70 can transport abnormal Tau to the lysosome/proteasome degradation system guided by BAG2/STUB1; They are important to maintain Tau’s internal balance (Demand et al., 2001; Young et al., 2016). In the process of lysosome degradation mediated by STUB1, the abnormal MAPT combined with HSC70 is ubiquitinated to a target protein containing multiple ubiquitin chains, which can be recognized by the proteasome and be refolded to the normal structure. This system will produce considerable abnormal MAPT if the coordination of the molecular network becomes disordered. When excessive abnormal Tau needs to be degraded, Tau’s proteasome degradation system is easy to block or loses function (Lee et al., 2013; Watanabe et al., 2020).

Overall, this study found the worsening of correlation of BAG2, HSC70, STUB1, and MAPT molecular network leads not only to a decrease in folding efficiency, but also to an elevated error rate. It may cause the formation of NFT and the changes of MMSE by contributing to the aggregation of abnormal tau.

## 5. Conclusion

This study revealed that the interaction consisting of BAG2, HSC70, STUB1, and MAPT play an important role in AD. By detecting their expression patterns, the clinical stages of AD can be diagnosed and determined. It facilitates epidemiological screening and early diagnosis of AD. The new method for predicting and detecting AD mentioned in this study does not require professional medical institutions and experienced experts. It can easily be rolled out to communities or hospitals, which will help reduce national and household health expenditures worldwide.

## Data availability statement

The datasets presented in this study can be found in online repositories. The names of the repository/repositories and accession number(s) can be found in the article/Supplementary material.

## Author contributions

XY, JL, and CP conceived and designed the study. WG collect and analyzed the data. WG and LY prepared the figures. XY and WG wrote the manuscript. XL, ZZ, and XP revised the manuscript. All authors contributed to the article and approved the submitted version.

## Conflict of interest

The authors declare that the research was conducted in the absence of any commercial or financial relationships that could be construed as a potential conflict of interest.

## Publisher’s note

All claims expressed in this article are solely those of the authors and do not necessarily represent those of their affiliated organizations, or those of the publisher, the editors and the reviewers. Any product that may be evaluated in this article, or claim that may be made by its manufacturer, is not guaranteed or endorsed by the publisher.

## Footnotes

^1^ https://www.uniprot.org/uniprotkb/O95816/entry#interaction
