# Supplementary material for: The relationship between protein modified folding molecular network and Alzheimer’s disease pathogenesis based on BAG2-HSC70-STUB1-MAPT expression patterns analysis
Source: Front Aging Neurosci. 2023 May 12;15:1090400. doi: 10.3389/fnagi.2023.1090400 (PMC10213342; doi:10.3389/fnagi.2023.1090400)
Supplement: Supplementary file 1 [file Table_1.DOCX]

Supplementary Material

Table1. The inner product matrix of control group

| **Control Group** | **BAG2** | **STUB1** | **HSPA8** | **MAPT** |
| --- | --- | --- | --- | --- |
| **BAG2** | 9 | -3.203384789 | -0.59999376 | 0.425514428 |
| **STUB1** | -3.203384789 | 9 | -3.584169993 | -6.756352832 |
| **HSPA8** | -0.59999376 | -3.584169993 | 9 | 6.011791459 |
| **MAPT** | 0.425514428 | -6.756352832 | 6.011791459 | 9 |

Table2. The inner product matrix of incipient group

| **Incipient Group** | **BAG2** | **STUB1** | **HSPA8** | **MAPT** |
| --- | --- | --- | --- | --- |
| **BAG2** | 7 | 0.504698415 | -0.582379377 | -0.826042755 |
| **STUB1** | 0.504698415 | 7 | -5.640187714 | -2.563322622 |
| **HSPA8** | -0.582379377 | -5.640187714 | 7 | 5.743505205 |
| **MAPT** | -0.826042755 | -2.563322622 | 5.743505205 | 7 |

Table3. The inner product matrix of moderate group

| **Moderate Group** | **BAG2** | **STUB1** | **HSPA8** | **MAPT** |
| --- | --- | --- | --- | --- |
| **BAG2** | 8 | -1.278814799 | 2.77512106 | 1.879185424 |
| **STUB1** | -1.278814799 | 8 | -6.072115731 | -0.887902238 |
| **HSPA8** | 2.77512106 | -6.072115731 | 8 | 3.02210915 |
| **MAPT** | 1.879185424 | -0.887902238 | 3.02210915 | 8 |

Table4. The inner product matrix of moderate group

| **Severe Group** | **BAG2** | **STUB1** | **HSPA8** | **MAPT** |
| --- | --- | --- | --- | --- |
| **BAG2** | 7 | -3.661255739 | 4.369722073 | 5.923803643 |
| **STUB1** | -3.661255739 | 7 | -4.901736977 | -5.728725374 |
| **HSPA8** | 4.369722073 | -4.901736977 | 7 | 5.689939016 |
| **MAPT** | 5.923803643 | -5.728725374 | 5.689939016 | 7 |

**Table 5. The details of Training Data in Neural Network**

| **Bag2** | **Stub1** | **Hspa8** | **Mapt** | **MMSE** | **PREDICTED MMSE** |
| --- | --- | --- | --- | --- | --- |
| 0.0857 | -2.2095 | 0.0239 | -2.2135 | 26 | 26.0552 |
| -0.1775 | 2.1138 | -0.3410 | 0.7857 | 18 | 15.5735 |
| -0.3709 | -0.1496 | -1.1686 | 0.3211 | 30 | 27.8028 |
| 0.6900 | -0.0040 | -0.0818 | 0.2433 | 11 | 13.5789 |
| -0.2513 | 0.5265 | -0.6162 | -0.0399 | 21 | 21.1346 |
| -0.8774 | -0.5564 | 0.6900 | -0.6022 | 14 | 13.7264 |
| 1.3355 | 2.0288 | -1.7756 | 0.8417 | 18 | 14.2610 |
| -1.0397 | -0.1688 | 0.3206 | -1.0767 | 26 | 26.1412 |
| -1.2467 | -0.0335 | 0.8421 | 1.1160 | 17 | 16.6053 |
| 0.8276 | -0.6988 | -0.4996 | -2.0285 | 20 | 19.8529 |
| 0.1708 | -0.9909 | -1.1631 | 0.7665 | 2 | 1.4495 |
| 0.2062 | -0.3360 | 0.0426 | -0.7627 | 11 | 11.7548 |
| -0.0621 | -0.8753 | -0.6661 | 0.8641 | 4 | 6.3619 |
| 0.4667 | -0.3574 | -0.2443 | 0.7712 | 28 | 22.6250 |
| -0.7845 | -0.5510 | 0.1402 | 0.4349 | 15 | 15.3970 |
| -0.7838 | -0.9111 | -0.0530 | -0.7678 | 2 | 2.4607 |
| -0.6054 | 1.5116 | 0.1540 | -0.7552 | 25 | 24.8829 |
| 0.0936 | -0.6742 | -0.5491 | 0.5611 | 15 | 13.2332 |
| -0.1655 | 0.6083 | -1.4164 | 1.4753 | 25 | 22.2791 |
| 0.1004 | -0.0746 | -1.0826 | 1.0545 | 7 | 14.5495 |
| 0.3678 | 1.8637 | -1.6691 | 0.7940 | 5 | 11.8467 |
| 1.9778 | 1.8383 | 0.9273 | -1.2507 | 29 | 29.4721 |
| -1.9982 | 0.1666 | -0.1139 | -0.2750 | 29 | 30.0522 |
| -0.3913 | 0.2160 | -0.3990 | -1.0349 | 26 | 25.6665 |

**Table 6. The details of Test Data in Neural Network**

| **Bag2** | **Stub1** | **Hspa8** | **Mapt** | **MMSE** | **PREDICTED MMSE** |
| --- | --- | --- | --- | --- | --- |
| 0.9053 | -0.6680 | 1.0729 | -0.2433 | 18 | 12.7085 |
| 0.7478 | -0.4507 | 0.0302 | 1.2471 | 28 | 24.6253 |
| 0.3086 | -0.3221 | 0.0999 | 2.0853 | 29 | 24.2084 |
| 1.2926 | 4.1993 | -0.0791 | -0.8279 | 24 | 24.8279 |
| -0.3990 | -0.6225 | 0.5088 | -0.0098 | 17 | 14.9680 |
| -0.0060 | -0.1347 | -0.7497 | -0.9917 | 26 | 23.1489 |

| Candidate Pathogenic Genes | Gene Symbol | Gene Name |
| --- | --- | --- |
| 200928_s_at' | RAB14 | RAB14, member RAS oncogene family |
| 201867_s_at' | TBL1X | transducin (beta)-like 1X-linked |
| 202933_s_at' | YES1 | v-yes-1 Yamaguchi sarcoma viral oncogene homolog 1 |
| 221021_s_at' | CTNNBL1 | catenin, beta like 1 |
| 204213_at' | PIGR | polymeric immunoglobulin receptor |
| 206278_at' | PTARF | platelet-activating factor receptor |
| 210128_s_at' | LTB4R | leukotriene B4 receptor |
| 214950_at' | IL9R | interleukin 9 receptor |
| 209643_s_at' | PLD2 | phospholipase D2 |
| 216916_s_at' | DLGAP2 | discs, large (Drosophila) homolog-associated protein 2 |
| 216059_at' | PAX3 | paired box 3 |
| 213301_x_at' | TRIM24 | tripartite motif containing 24 |
| 221070 _s_at' | KIAA1967 | KIAA1967 |
| 208111_at' | AVPR2 | arginine vasopressin receptor 2 |
| 217486_s_at' | ZDHHC17 | zinc finger, DHHC-type containing 17 |
| 203643_at' | ERF | Ets2 repressor factor |
